# IGF-1R tyrosine kinase inhibitors and Vitamin K1 enhance the antitumor effects of Regorafenib in HCC cell lines

**DOI:** 10.18632/oncotarget.21403

**Published:** 2017-09-30

**Authors:** Maria Grazia Refolo, Rosalba D’Alessandro, Catia Lippolis, Nicola Carella, Aldo Cavallini, Caterina Messa, Brian Irving Carr

**Affiliations:** ^1^ Laboratory of Cellular and Molecular Biology, Department of Clinical Pathology, National Institute of Gastroenterology, “S. De Bellis” Research Hospital, Castellana Grotte, BA, Italy; ^2^ Visiting Professor, Program for Targeted Experimental Therapeutics, Izmir Biomedicine and Genome Center, Dokuz Eylul University, Izmir, Turkey

**Keywords:** combination therapy, synergism, regorafenib, vitamin K1, insulin-like growth factor receptor

## Abstract

The recent RESORCE trial showed that treatment with Regorafenib after Sorafenib failure provided a significant improvement in overall survival in HCC patients. Preclinical and clinical trial data showed that Regorafenib is a more potent drug than Sorafenib. In this study we aimed at improving Regorafenib actions and at reducing its toxicity, by targeting parallel pathways or by combination with Vitamins K (VKs). We investigated the effects of Regorafenib administrated at low concentrations and in combination with either VK1 and/or with GSK1838705A or OSI-906, two IGF1-R inhibitors, on HCC cell growth and motility. Our results showed that both IGF1-R inhibitors potentiated the antiproliferative and pro-apoptotic effects of Regorafenib and/or VK1 in HCC cell lines. Moreover we provide evidence that the combined treatment with IG1-R antagonists and Regorafenib (and/or VK1) also caused a significant reduction and depolymerization of actin resulting in synergistic inhibition exerted on cell migration. Thus, simultaneous blocking of MAPK and PI3K/Akt cascades with IGF1-R inhibitors plus Regorafenib could represent a more potent approach for HCC treatment.

## INTRODUCTION

Hepatocellular carcinoma (HCC) is the second most frequent cause of cancer-related death worldwide [1]. The multikinase inhibitor Sorafenib is the only systemic drug approved for the first-line treatment of advanced HCC. Although Sorafenib treatment provides a significant improvement in overall survival, a high percentage of Sorafenib-treated patients experience disease progression and major toxicity [2].

Recently, the RESORCE trial showed that treatment with Regorafenib (Fluoro-Sorafenib) provided a significant improvement in overall survival in patients with HCC who had disease progression during first-line treatment with Sorafenib. Median overall survival was 10.6 months in the Regorafenib group compared with 7.8 months in those receiving placebo, representing a significant reduction in the risk for death [3]. The significance of this is that Regorafenib exerted responses and survival benefits even in Sorafenib resistant patients. Thus, these two drugs must be non-identical in their actions.

Regorafenib is also an oral multikinase inhibitor, that has action against cRAF/RAF-1, B-RAF, PDGF and c-Kit [4–6]. However, despite the availability of these targeted kinase inhibitors, drug toxicity and resistance and subsequent disease relapse remain a problem in HCC management. Several studies have aimed at improving the actions of these systemic drugs and at reducing their toxicity, such as by targeting parallel pathways [7] or by combination with Vitamins K (VKs) [8–9]. VKs are fat-soluble vitamins with minimal toxicity, that play a major role not only in blood coagulation and bone metabolism [10], but also have anti-tumor activity in different experimental tumor types [11–13]. Many mitogens have been shown to be important for HCC growth. Amongst them there is IGF1 [14] and we have shown that inhibition of IGF1-R antagonizes HCC cell lines growth [15]. In the current work we extend these previous observations to show that addition of IGF1-R inhibition enhances the effects of Regorafenib as well as its combination with VK, and in addition permits the use of lower Regorafenib concentrations, that might result in lower toxicity.

## RESULTS

### GSK1838705A and OSI-906 potentiate the antiproliferative effects of Regorafenib and/or VK1 in HCC cell lines

**Figure 1:**
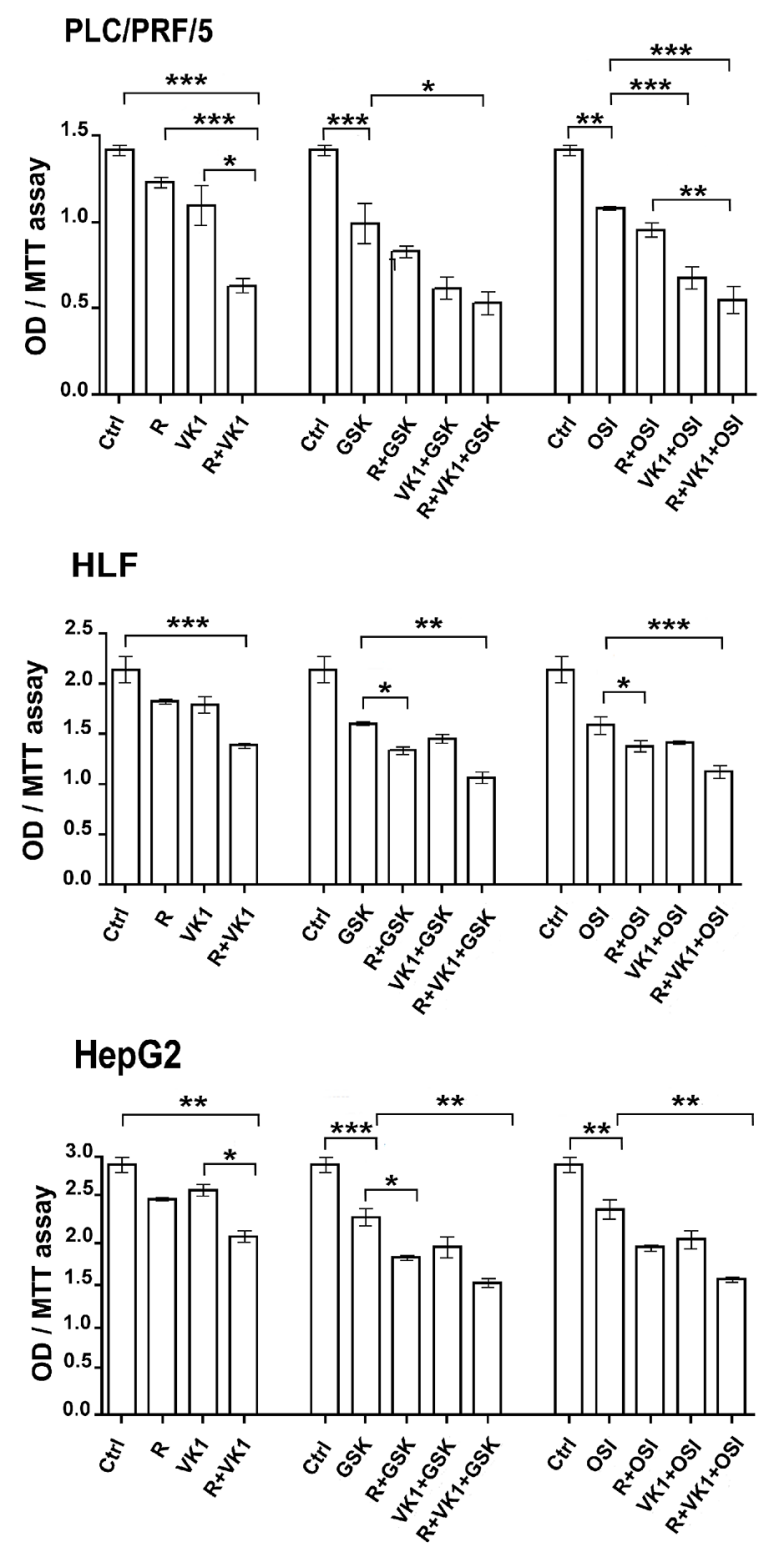
GSK1838705A and OSI-906 potentiate the antiproliferative effects of Regorafenib and/or VK1 in HCC cells Cells were cultured with 1μM (PLC/PRF/5) or 0.1μM (HepG2) Regorafenib, 25μM VK1, 4μM GSK1838705A and 0.5μM OSI-906 administrated singularly or in combination. MTT assay was performed after 48 h. The results of three independent experiments are expressed as means ± SD. ^*^p < 0.05; ^**^p < 0.001; ^***^p < 0.0001.

We initially examined whether addition of IGF1-R inhibition could enhance Regorafenib effect. A range of concentrations was examined for each of the drugs that were studied (data not shown) and the lowest concentration of each drug that had a biological effect was subsequently used in all assays. PLC/PRF/5, HepG2 or HLF cells were cultured in presence of Regorafenib and/or VK1. Cells that were treated with Regorafenib and/or VK1 were also cultured with or without GSK1838705A or OSI-906, two different IGF1-R inhibitors. Cell proliferation was evaluated after 48 h by MTT assay. Addition of VK1 exerted a potent inhibitory effect on cell growth in all the combinations of drugs analyzed. In particular, we found that VK1 enhanced the inhibitory effect of Regorafenib. Furthermore, the addition of this natural compound permitted the growth inhibition by Regorafenib at a concentration that was ten times lower than its IC50. In addition, this inhibitory effect was further potentiated by the combination with either GSK1838705A or OSI-906 (Figure 1). A combination index (CI) was computed for these combinations. Average CI values computed between the different cell lines were 0.5 and 0.7 for GSK1838705A/Regorafenib/VK1 and OSI-906/Regorafenib/VK1 combinations respectively. These values were found to be well below the line of additivity (CI ≤ 1), showing that synergy was involved in these drug interactions (data not shown).

### AFP response

**Figure 2:**
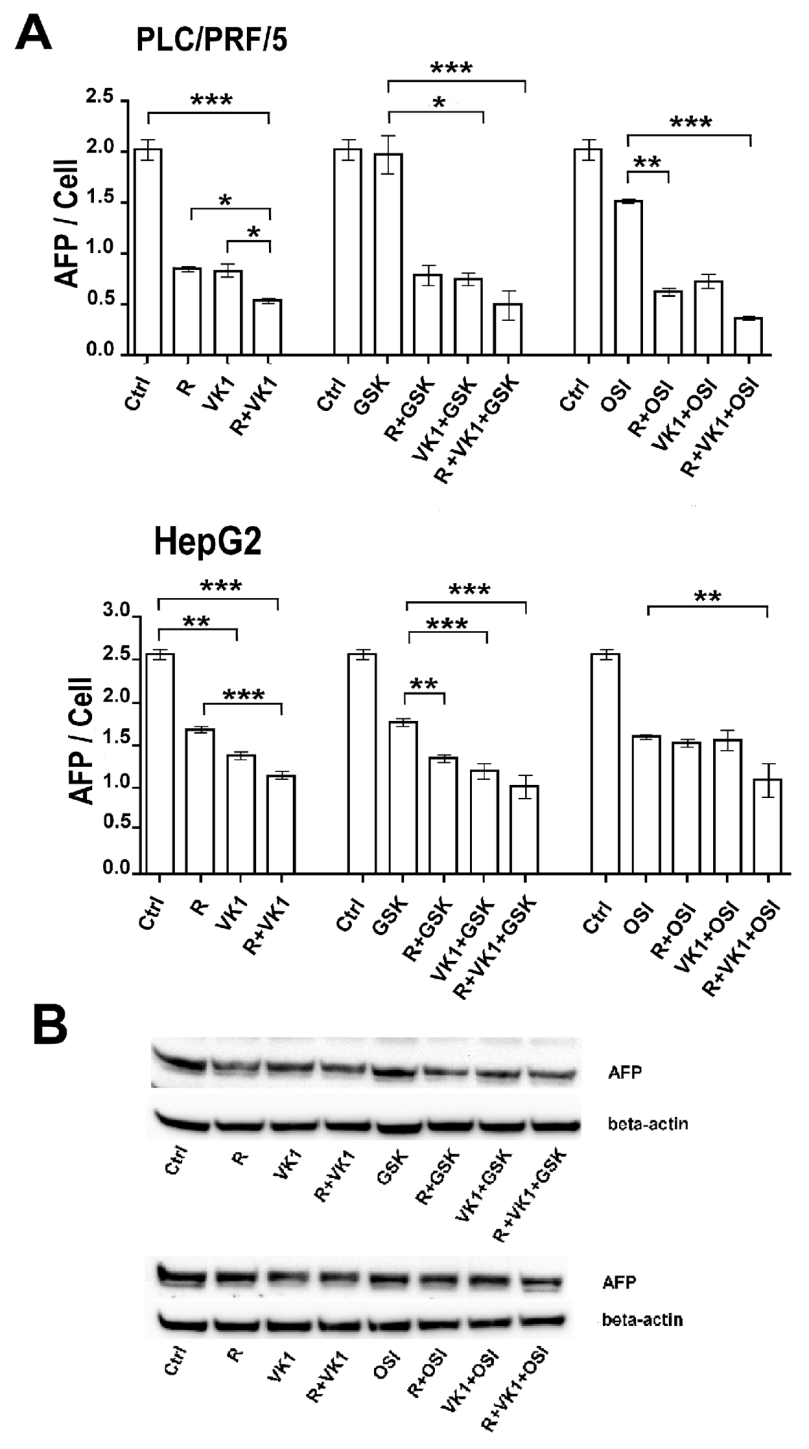
GSK1838705A and OSI-906 potentiate the Regorafenib/VK1-mediated inhibition of AFP secretion **(A)** AFP levels in the cell culture medium of PLC/PRF/5 and HepG2 cell lines and the number of viable cells after treatment of 48 h with 1μM (PLC/PRF/5) or 0.1μM (HepG2) Regorafenib, 25μM VK1, 4μM GSK1838705A and 0.5μM OSI-906 administrated singularly or in combination. **(B)** Western blotting analysis of cellular AFP in PLC/PRF/5 cells. The experiments were repeated three times. The results of three independent experiments are expressed as means ± SD. ^*^p < 0.05; ^**^p < 0.001; ^***^p < 0.0001.

Since AFP is an important clinical tumor marker for HCC growth and aggressiveness, we examined the effects of GSK1838705A and OSI-906 on Regorafenib/VK1-mediated inhibition of AFP secretion levels after 48 h, using the two AFP-secreting human HCC cell lines, PLC/PRF/5 and HepG2. Despite the significant reduction in the medium AFP levels in cells treated with the combination of Regorafenib/VK1, the enhancement exerted by further addition of IGF1-R inhibitors was weak (Figure 2A).

These results were confirmed also by Western blot analysis for cellular AFP expression (Figure 2B).

### GSK1838705A and OSI-906 potentiate the pro-apoptotic effects of Regorafenib and/or VK1 in HCC cell lines

**Figure 3:**
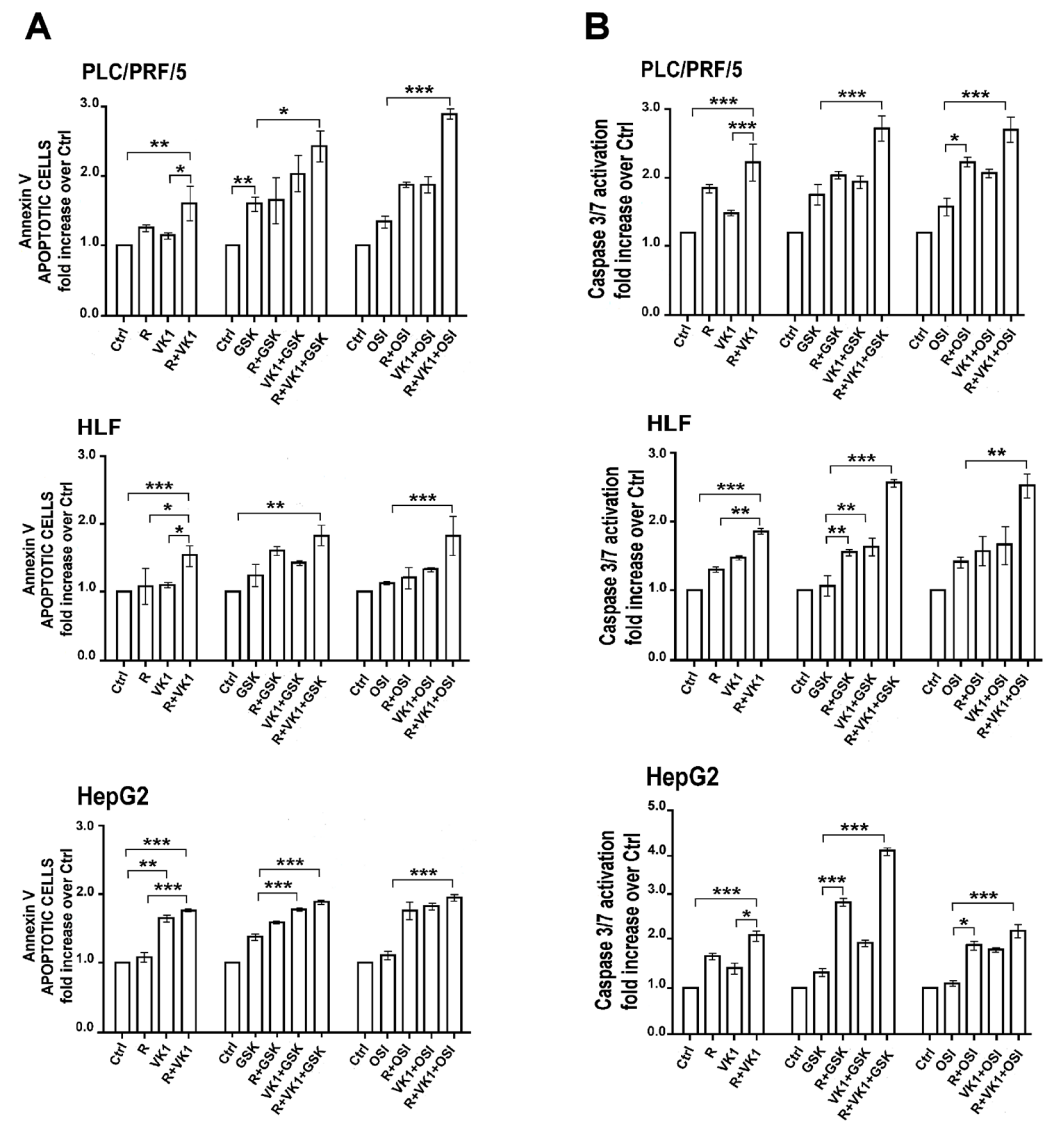
GSK1838705A and OSI-906 potentiate the pro-apoptotic effects of Regorafenib and/or VK1 in HCC cell lines Cells were cultured with 1μM (PLC/PRF/5) or 0.1μM (HepG2) Regorafenib, 25μM VK1, 4μM GSK1838705A and 0.5μM OSI-906 administrated singularly or in combination. Muse Annexin V **(A)** and Muse Caspase-3/7 **(B)** Cell Assays were performed after 48 h. The results of three independent experiments are expressed as means ± SD. ^*^p < 0.05; ^**^p < 0.001; ^***^p < 0.0001.

We next investigated the effects of GSK1838705A or OSI-906 on Regorafenib/VK1-mediated apoptosis after 48 h, using the same experimental conditions as for the growth experiments. VK1 added simultaneously to Regorafenib (0.1μM for HepG2 and 1μM for both PLC/PRF/5 and HLF) caused an average increase of 44% in cellular Annexin V compared with Regorafenib treated cells. The percentage of apoptotic cells was further increased of 74% with GSK1838705A and of 100% with OSI-906 compared with the combination Regorafenib and VK1 (Figure 3A). These findings were confirmed by the evaluation of apoptotic status based on Caspase-3 and -7 activation analyzed by the Muse Caspase-3/7 assay (Figure 3B).

### GSK1838705A and OSI-906 potentiate the inhibitory effects of Regorafenib and/or VK1 in HCC cell migration and on actin polymerization

**Figure 4:**
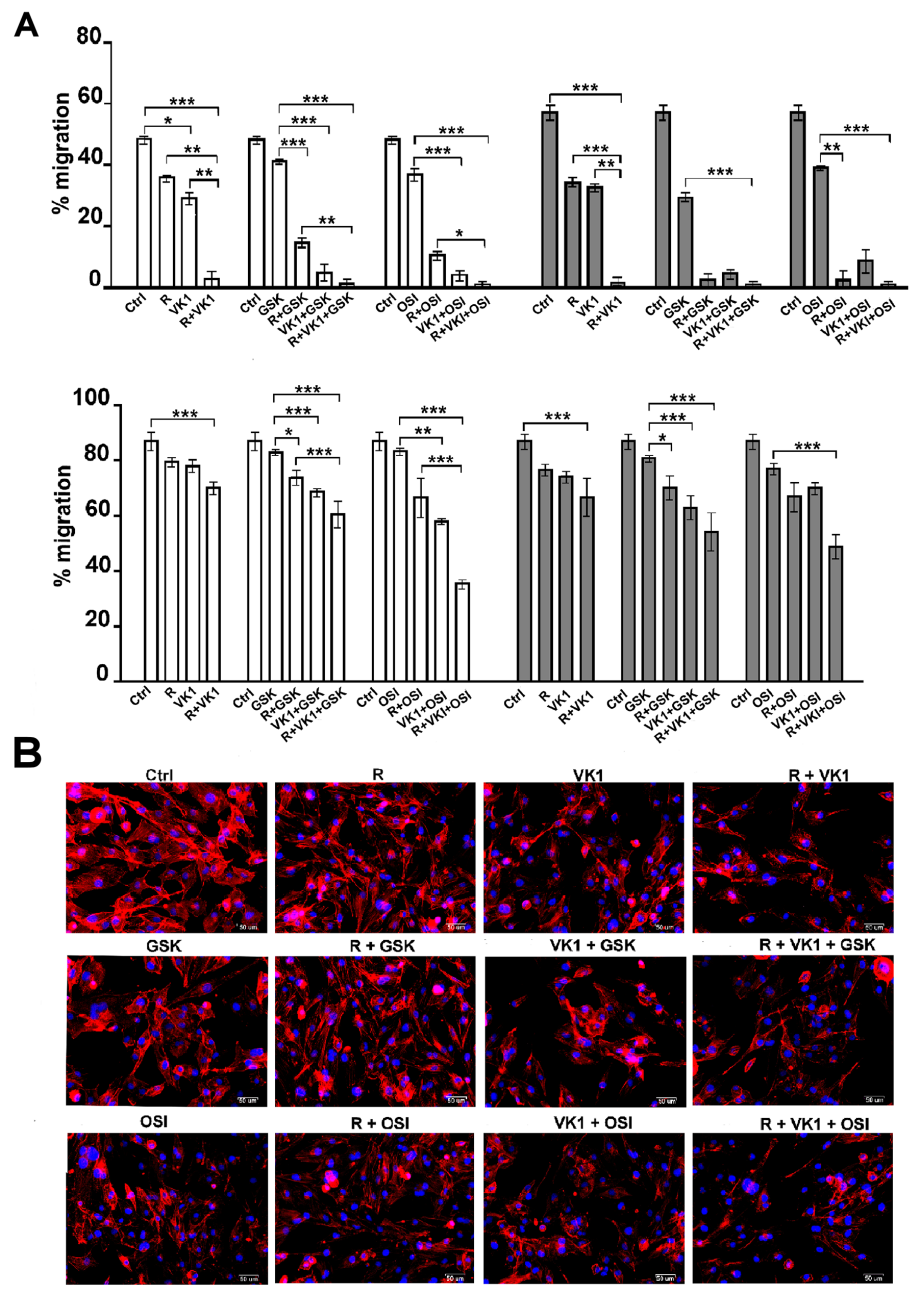
GSK1838705A and OSI-906 enhance the inhibitory effects of Regorafenib and/or VK1 on depolymerization of actin cytoskeleton and cell migration **(A)** Migration assay was performed in PLC/PRF/5 and HLF cells seeded on collagen I and fibronectin coated wells and treated as described. The microscopic analysis was assessed at the time T0 and T2 (after 48 h). The values were expressed as percentage of migration, where 100% represents the detection zone completely closed. The results of three independent experiments, expressed as mean±SD, are plotted in the relative graph. ^*^p < 0.05; ^**^p < 0.001; ^***^p < 0.0001. **(B)** HLF cells stained with DyLight 554 Phalloidin after treatment with 1μM Regorafenib, 25μM VK1, 4μM GSK1838705A and 0.5μM OSI-906 administrated singularly or in combination. Scale bar: 50μm.

To test the effects of GSK1838705A and OSI-906 on Regorafenib/VK1-mediated inhibition of cell migration, cells were seeded onto Oris plates and treated with drugs according to the experimental conditions above. Cell migration assay was performed on wells coated with Collagen I and Fibronectin matrix and microscopic analysis was assessed both immediately after the stoppers removal (T0) and different later times, in the graphs were reported the percentage of migration after 48 h. We found that the inhibition exerted by Regorafenib on migration was strongly potentiated with either VK1 and IGF1-R inhibitors and that these effects were independent of the matrix used. Interestingly, all the combination treatments showed synergistic effects (CI < 1, data not shown) in driving the inhibition of cell migration compared to the single agent (Figure 4A). We next examined whether treatment of cells with these drug combinations affected the actin cytoskeleton. Cells that were stained with DyLight 554 Phalloidin revealed that both Regorafenib and VK1, administrated for 24 h alone or in combination with GSK1838705A or OSI-906, caused significant reduction and depolymerization of actin in the cells on Fibronectin matrix. This resulted in a loss of F-actin fibers in the cytoplasm and their redistribution around the nucleus, whereas in control cells actin fibers were diffusely distributed in the cytoplasm (Figure 4B).

### GSK1838705A and OSI-906 potentiate the inhibition by Regorafenib of MAPK and PI3K/Akt signaling

**Figure 5:**
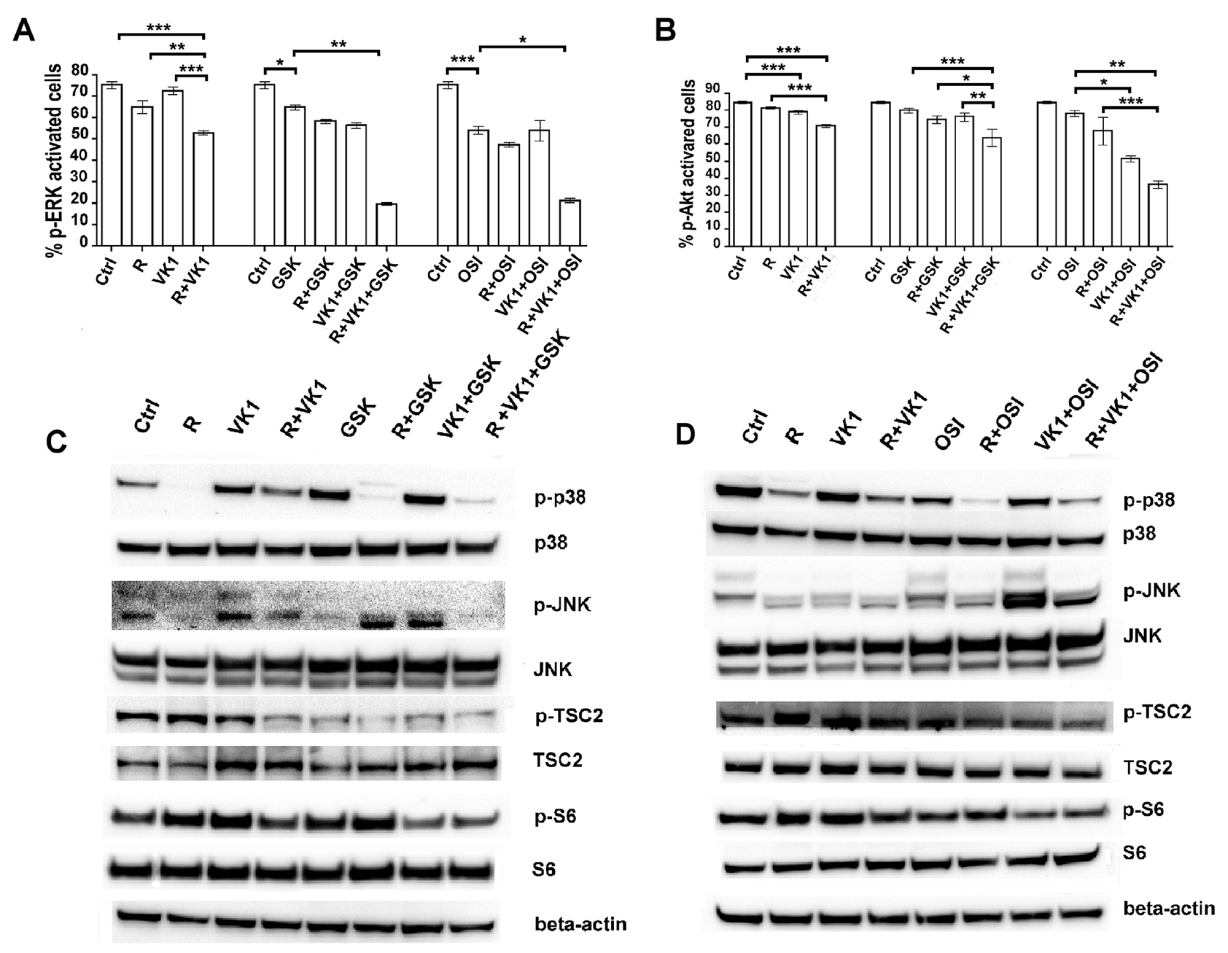
Effects of the combination Regorafenib/VK1 and IGF1-R inhibitors on the modulation of MAPK and PI3K/Akt signaling PLC/PRF/5 cells were cultured with 1μM Regorafenib, 25μM VK1, 4μM GSK1838705A and 0.5μM OSI-906 administrated singularly or in combination. **(A)** The Muse MAPK Activation Kit was used to evaluate the MAPK phosphorylation relative to the total MAPK expression after 15 min. The results of three independent experiments, expressed as mean±SD, are plotted in the relative graph. **(B)** The Muse PI3K Activation dual detection Kit was used to detect the Akt phosphorylation (Ser473) relative to the total Akt expression after 24 h. The results of three independent experiments, expressed as mean±SD, are plotted in the relative graph. ^*^p < 0.05; ^**^p < 0.001; ^***^p < 0.0001. **(C-D)** Western Blot showing the expression levels of some proteins involved in MAPK and PI3K/Akt pathways after 48 h of single or combined treatments.

We have previously shown that Regorafenib and GSK1838705A decrease the levels of p-IGF1-R (15) and therefore now wished to examine the downstream signaling. Cells treated with Regorafenib and/or VK1 were additionally cultured with or without GSK1838705A or OSI-906. MAPK phosphorylation was evaluated relative to the total MAPK expression by Muse MAPK Activation Dual Detection Kit. Addition of VK1 to the cultures caused an additional decrease of 18% in pERK levels, compared with Regorafenib alone (12%) used at low concentrations (Figure 5A). Both IGF1-R antagonists further enhanced this inhibitory effect when either was added in combination with Regorafenib and VK1, and caused a decrease of 62% in pERK levels, using GSK1838705A and a decrease of 60%, using OSI-906. Using Muse PI3K Activation dual detection Kit, we investigated the activation of PI3K/Akt pathway in the same experimental conditions. We found that neither Regorafenib nor VK1 had any effect on the percentage of p-Akt (Ser473) activation, but when these two drugs were added in combination, we found a decrease of 15% in p-Akt levels with respect to untreated cells. An even greater reduction in p-Akt was obtained by further addition of either of the IGF1-R inhibitors. GSK1838705A and OSI-906, which caused a further decrease of 10% and 50% respectively in p-Akt levels when they were added in combination with Regorafenib and VK1 (Figure 5B). Western Blot analysis of cell lysates in the same experimental conditions was performed to measure the phosphorylation levels of several proteins involved in MAPK and PI3K signaling. The expression levels of p-p38 and p-JNK, which are involved in MAPK signaling, were decreased in cells treated both with Regorafenib/VK1 and with Regorafenib/VK1/IGF1-R inhibitor combinations. By contrast, the expression levels of p-TSC2 and p-S6, which are involved in PI3K/Akt signaling, were significantly reduced only in Regorafenib/VK1 treated cells when they were also treated with IGF1-R inhibitors (Figure 5C–5D).

## DISCUSSION

The outcome of the recent RESORCE trial was that Regorafenib treatment provided survival benefit in patients with HCC, after progression on Sorafenib treatment [3]. These results suggest that the sequential use of molecules with tyrosine-kinase inhibitor activity can provide a significant improvement in overall survival. Preclinical and trial data highlighted that Regorafenib with respect to its analog Sorafenib, is a more potent drug, that is may be able to inhibit a distinct set of kinases, including the angiogenic and stromal receptors VEGFR-1-3, TIE-2, FGFR1 and PDGF-β as well as the oncogenic receptors KIT and RET [5, 16–18]. However, drug toxicity and the presence of resistance mechanisms in the majority of patients [19] remain a problem in HCC treatment. Our previous *in vitro* studies suggested that microenvironmental factors can promote resistance to multikinase inhibitors drugs or chemotherapy [15, 20]. The studies of the resistance mechanisms to Sorafenib have revealed that in tumors with acquired resistance, there is an over expression of IGF1 and its major down-stream pathway PI3K-Akt [21]. Moreover, we previously reported that platelet-associated IGF1 antagonized Regorafenib-mediated growth, migration and invasion inhibition, as well as the drug-mediated induction of apoptosis in HCC cells *in vitro* [15], suggesting common escape mechanisms for both Sorafenib and Regorafenib. Hence, simultaneous blocking of MAPK and PI3K/Akt cascades with IGF1-R inhibitors and Regorafenib could represent a crucial approach for HCC treatment. The strategies to block IGF1-R include three different classes of drugs: monoclonal anti-IGF1-R antibodies, small molecules tyrosine kinase inhibitors (TKIs) and IGF ligand antibodies. Currently, several anti-IGF1-R antibodies and TKI molecules have demonstrated that these targeting approaches can induce strong anti-tumor activities and are under clinical investigation. Among TKIs, OSI-906 and BMS754807 are the most specific and act on the intra-cellular domain of IGF1-R. Others, such as GSK1838705A, act on the extra-cellular domain [22]. Moreover, we found that GSK1838705A strongly enhanced Regorafenib action [15], showing that the IGF1-R and PI3K/Akt/mTOR signaling pathways are associated with HCC biology [15, 23, 24]. Since both Sorafenib and Regorafenib have multiple toxicities and a large percent of patients need to be dose-reduced or stop taking the drugs, our previous studies had underlined that the combined administration of both VK1 and Sorafenib drastically reduced the drug dosage that was required for growth inhibition in several HCC cell lines [9, 11, 33]. Moreover, non-toxic VK1 could inhibit any recovery of growth and migration in Regorafenib pre-treated HCC cells [25]. Overall this knowledge led us to investigate the effects of Regorafenib administrated at low concentrations and in combination with either VK1 and the IGF1-R inhibitors on HCC cells growth and motility. In the present study we found that VK1 enhanced the inhibitory effect of Regorafenib on cell proliferation. Furthermore, the addition of this natural compound allowed the use of a concentration of Regorafenib that was ten times lower than its IC50. The inhibitory effect was further potentiated by the combination with either of the IGF1-R inhibitors used, GSK1838705A and OSI-906. The combination indexes (CI) computed for these drug combinations were found to be well below the line of additivity (CI ≤ 1), showing that drug synergy was likely involved in these drug interactions. Since AFP is an important clinical tumor marker for HCC growth and aggressiveness, we examined the effects of GSK1838705A and OSI-906 on Regorafenib/VK1-mediated inhibition of AFP secretion levels in HCC cell lines. These experiments revealed a significant reduction in the medium AFP levels in cells treated with the combination of Regorafenib and VK1, whereas the enhancement exerted by further addition of IGF1-R inhibitors was weak, suggesting a major involvement of MAPK signaling. Synergistic effects obtained by combining GSK1838705A or OSI-906 with Regorafenib/VK1 were also clearly visible in the induction of apoptosis. VK1 added simultaneously to cells with Regorafenib caused an average increase of 44% compared with Regorafenib treated cells, and this percentage was further enhanced by the addition of GSK1838705A (74%) or OSI-906 (100%).

Previous studies have shown that IGF1 had a protective effect on Sorafenib- or VK1-mediated reduction and depolymerization of actin cytoskeleton, with ensuing effects on cell motility [15, 23, 24]. Here, we provide evidence that the combined treatment with IGF1-R antagonists and Regorafenib and/or VK1 caused a significant reduction and depolymerization of actin, resulting in synergistic inhibition of cell migration. These data are consistent with those of Western Blot analysis, in which we evaluated the phosphorylation level of some of the principal proteins involved in the MAPK and PI3K/Akt signaling cascades which are involved in cell proliferation, oncogenesis, differentiation, inflammation and stress responses [26].

These experiments are the first to show the enhancement of the growth-inhibitory actions of Regorafenib by the totally non-toxic (to humans) vitamin K [34], as well as by IGF1-R inhibitors. Given the considerable toxicity in patients of both Sorafenib and Regorafenib, combinations with other agents that can enhance its effectiveness and thus permit lower clinical doses to be used, are of potential clinical interest.

Overall these results indicate that therapies based on the combination of agents with additive or synergistic activity could represent new therapeutic strategies against HCC and might permit the use of lower and less toxic Regorafenib doses.

## MATERIALS AND METHODS

### Cells and drugs

PLC/PRF/5, HepG2 and HLF human HCC cell lines were purchased from the National Institute of Biomedical Innovation JCRB Cell Bank (Osaka, Japan). The culture medium was Dulbecco’s Modified Eagle’s Medium (DMEM). All cell culture components were purchased from Sigma-Aldrich (Milan, Italy). Regorafenib was gifted from Bayer Corp (West Haven, CT USA ); vitamin K1 was purchased from International Medication Systems, Limited (So. El Monte, CA, USA), GSK1838705A and S1091 Linsitinib (OSI-906) from Selleckchem (Houston, TX, USA).

### Cell culture

PLC/PRF/5, HepG2 and HLF cell lines were cultured in DMEM in monolayer culture, and supplemented with 10% fetal bovine serum (FBS), 100U/ml penicillin, 100μg/ml streptomycin, and incubated at 37°C in a humidified atmosphere containing 5% CO_2_ in air. Since PLC/PRF/5 cells express high affinity IGF1-R that may mediate the stimulatory effects of exogenous IGF1 [27], we chose to investigate the MAPK activation and PI3K/Akt signaling markers only in this cell line.

### Cell proliferation and drug synergy evaluation

The cells were cultured in medium containing different concentrations of Regorafenib (1, 2.5, 5μM) and/or VK1 (6, 12, 25μM) in presence of GSK1838705A (2, 4, 6μM) or OSI-906 (0.13, 0.5, 1μM). After 48h of incubation, the proliferative response was estimated by colorimetric 3-(4,5 di-methylthiazol-2-yl)-2,5-diphenyltetrazolium bromide (MTT) test. The trypan blue exclusion test was used to evaluate cell viability. Each experiment was performed in triplicate and repeated three times.

The potential synergistic, additive or antagonistic effects of Regorafenib or VK1 plus GSK1838705A or OSI-906 used in combination were assessed experimentally and computationally using methods as described by Chou and Chou and Talalay [28, 29], implemented in CompuSyn software (Biosoft, UK). The Chou and Talalay approach takes into account drug potency as well as the relationship between dose and response for each drug. Results are reported as the Combination Index (CI). Values of CI < 1, CI ± 1, and CI > 1 imply synergy, additivity, and antagonism, respectively. An isobologram, based on an extension of the Lowe additivity model is also provided [30]. Similar to the CI values, values on the isobologram below the line of additivity represent combinations where synergy is present, values close to the line would represent additivity, and values above the line represent antagonistic effects. Moreover the Dose Reduction Index (DRI) was computed using CompuSyn software, this value denotes how many fold of dose reduction is allowed for each drug due to synergism when compared with the dose of each drug alone. In all the subsequent combined treatments each drug was used at the concentration showing above CI < 1, in particular cells were cultured with 1μM (PLC/PRF/5) or 0.1μM (HepG2) Regorafenib, 25μM VK1, 4μM GSK1838705A and 0.5μM OSI-906 administrated singularly or in combination.

### AFP measurement

The cells were cultured as described in the proliferation paragraph. Medium AFP levels were measured by use of an automated system (UniCel Integrated WorkstationsDxC 660i, Beckman–Coulter, Fullerton, CA) using a chemiluminescent immunometric method. Sample measurements over the calibration range were automatically re-analyzed according to constructor’s instructions.

### Apoptosis assay

The cells were cultured as described in the proliferation paragraph. Two different apoptosis experiments were performed. Flow cytometry technology (Muse Cell Analyzer, Millipore, Darmstadt, Germany) was used to detect the fluorescent signal emitted by dye conjugated antibodies. The Muse Annexin V/Dead Cell Assay Kit (Millipore, Darmstadt, Germany) for quantitative analysis of live, early/late apoptotic and dead cells was used with a Muse Cell Analyzer. Briefly, the assay utilizes Annexin V to detect PS on the external membrane of apoptotic cells. A dead cell marker (7-AAD) is also used. The cells were then processed as described in the user’s guide. Moreover, the simultaneous evaluation of apoptotic status based on Caspase-3 and −7 activation and cellular plasma membrane permeabilization (cell death) was analyzed by the Muse Caspase-3/7 kit (Millipore). The assay provides relative percentage of cells that are live, early/late apoptotic or dead. Cells were processed according to the user’s guide.

### Migration assay

The cells were cultured as described in the proliferation paragraph. The migration assay was performed by Oris Cell Migration Assay (Platypus Technologies, Madison USA) that provided wells coated with Collagen I, Fibronectin or tissue culture treated. This TriCoated sytem permits the evaluation of the best surface coating for HCC cell lines used. Briefly, the cells seeded onto Oris plates have been to adhere on their surface except in the circle covered with a stopper (detection zone) and were subjected to different drug treatments. When the stoppers have been removed the cells started to migrate into the detection zone. Cells were examined microscopically throughout the incubation period to monitor progression of migration in the detection zone. Migration time was depended upon cell type, type of coating and specific drug treatment. Photographs were taken of each well immediately after the stoppers removal (T0) and after 24 h (T1), 48 h (T2) and 72 h (T3).

The values were expressed as percentage of migration, with 100% being when the detection zone was completely closed. The results were representative of three independent experiments. The relative graphs were created with GraphPad Prism 5.0 software.

### Immunofluorescence

PLC/PRF/5 and HLF cells were cultured for 24 h in 1% FBS medium and then were trypsinized, resuspended in 1% FBS medium and seeded in 96 multi-well plates. Experiments were performed as described previously [31]. Specifically, cell monolayers in 96 multi-well plates were fixed with Cytofix fixation buffer (BD biosciences, Milan, Italy) 10 minutes at room temperature and quenched in 0.1M glycine, then permeabilized for 20 min in PBS containing 0.1% Triton X-100, blocked for 30 min at room temperature in BlockAid solution (Life technologies, Eugene, OR, USA) and immunolabeled in humidified dark chamber for 2 h with DyLight 554 Phalloidin (Cell Signaling, Beverly, MA, USA). Nuclei were stained with DAPI for 10 min in the dark. After rinsing with PBS, images were acquired with ZOE fluorescent cell imager (Bio-Rad, Milan, Italy).

### MAPK activation assay

Muse MAPK Activation Dual Detection Kit (Millipore, Darmstadt, Germany), including a phospho-specific antiphospho-Erk1/2 (Thr202/Tyr204, Thr185/Tyr187)-Phycoerythrin and an anti-Erk1/2-PECy5 conjugated antibody, was used to measure total levels of Erk. This two-color kit is designed to measure the extent of MAPK phosphorylation relative to the total MAPK expression in the cell population processed as described in the user’s guide. The levels of both the total and phosphorylated protein can be measured simultaneously in the same cell, resulting in a normalized measurement of MAPK activation after stimulation. PLC/PRF/5 cell line was cultured in presence of the indicated concentrations of Regorafenib, VK1, GSK1838705A and OSI-906 alone or in combination for 15 min. Results were representative of three independent experiments. The relative graphs were created with GraphPad Prism 5.0 software.

### PI3K activation assay

Muse PI3K Activation dual detection Kit was used to detect the fluorescent signal emitted by dye conjugated antibodies, a specific anti-phospho-Akt (Ser473), Alexa Fluor 555, and an Akt,PECy5 conjugated antibody to measure total levels of Akt. This two color kit is designed to measure the extent of Akt phosphorylation relative to the total Akt expression in any given cell population processed as described in the user’s guide. The levels of both the total and phosphorylated protein can be measured simultaneously in the same cell, resulting in a normalized measurement of PI3K activation after stimulation. PLC/PRF/5 cell line was cultured in presence of the indicated concentrations of Regorafenib, VK1, GSK1838705A and OSI-906 alone or in combination for 24 h. Results were representative of three independent experiments. The relative graphs were created with GraphPad Prism 5.0 software.

### Western blot

MAPK and PI3K/Akt signaling markers were evaluated in PLC/PRF/5 cells treated for 48 h as indicated above. Western Blot analysis was performed as previously described [32]. Briefly, cells were washed twice with cold PBS and then lysed in RIPA buffer (Sigma-Aldrich, Milan; Italy). After quantization of protein concentration, equal amount of protein (50μg) were resolved on SDS–PAGE and transferred to polyvinyldifluoride (PVDF) filters. The blots were blocked with 5% (w/v) nonfat dry milk for 2 h at room temperature and then probed with primary antibody overnight at 4°C. The primary antibodies were directed against the following proteins: IGF1-R and phospho-IGF1-R (P-IGF1-R tyr1316), p38 and phospho-p38 (P-p38 thr180/tyr182), JNK and phospho-JNK (P-JNK thr183/tyr185), TSC2 and phospho-TSC2 (Ser939), S6 and phospho-S6 (Ser235/236), AFP and β-actin (Cell Signaling, Beverly, MA, USA). After three washes, incubation was followed by the reaction with horseradish peroxidase-conjugated secondary antibody for 1 h at room temperature. The immunoreactive bands were visualized and analyzed using enhanced chemiluminescence detection reagents, according to the manufacturer’s instructions, and chemiluminescence detection system (ChemiDoc XRS apparatus and software, Bio-Rad, Milan, Italy).

### Statistical analysis

GraphPad Prism 5.0 software (La Jolla, CA, USA) was used for all statistical analysis. The significance of difference between the groups was assessed by ANOVA, followed by Kruskal-Wallis nonparametric test. *P*<0.05 was considered statistically significant. All experiments were performed in triplicate and were replicated three times. Data are presented as mean ± standard deviation (SD).
